# Myths, Artifacts, and Fatal Flaws: Identifying Limitations and Opportunities in Vitamin C Research

**DOI:** 10.3390/nu5125161

**Published:** 2013-12-16

**Authors:** Alexander J. Michels, Balz Frei

**Affiliations:** Linus Pauling Institute, 307 Linus Pauling Science Center, Oregon State University, Corvallis, OR 97331, USA; E-Mail: balz.frei@oregonstate.edu

**Keywords:** vitamin C, ascorbic acid, cell culture, animals, human, study design

## Abstract

Research progress to understand the role of vitamin C (ascorbic acid) in human health has been slow in coming. This is predominantly the result of several flawed approaches to study design, often lacking a full appreciation of the redox chemistry and biology of ascorbic acid. In this review, we summarize our knowledge surrounding the limitations of common approaches used in vitamin C research. In human cell culture, the primary issues are the high oxygen environment, presence of redox-active transition metal ions in culture media, and the use of immortalized cell lines grown in the absence of supplemental ascorbic acid. Studies in animal models are also limited due to the presence of endogenous ascorbic acid synthesis. Despite the use of genetically altered rodent strains lacking synthesis capacity, there are additional concerns that these models do not adequately recapitulate the effects of vitamin C deprivation and supplementation observed in humans. Lastly, several flaws in study design endemic to randomized controlled trials and other human studies greatly limit their conclusions and impact. There also is anecdotal evidence of positive and negative health effects of vitamin C that are widely accepted but have not been substantiated. Only with careful attention to study design and experimental detail can we further our understanding of the possible roles of vitamin C in promoting human health and preventing or treating disease.

## 1. Introduction

Ascorbic acid, the reduced form of vitamin C, is an essential component of the human diet. Small amounts of ascorbic acid can prevent the deficiency disease, scurvy, while accumulation of high levels of ascorbate in plasma and tissues may protect against oxidative damage and limit inflammation. However, ascorbic acid is unlike many other vitamins owing in part to its unique redox chemistry. In addition, the “tight control” of vitamin C status and metabolism in the body, along with biological effects of supplementation that may differ between animals that can synthesize ascorbate *versus* species that cannot synthesize ascorbate, like humans, set it apart in the micronutrient field [1,2,3]. Thus, many common research practices that are sufficient for the study of other vitamins and minerals are often inadequate for the study of vitamin C, leaving the specific challenges to the design and execution of experiments utilizing ascorbic acid underappreciated. Indeed, there are many examples of supplementation studies making poor assumptions and drawing mistaken conclusions that have persisted in the vitamin C literature. Although several landmark discoveries have broadened our understanding of vitamin C’s role in human biology, the research is still plagued by a host of myths, artifacts, and flawed scientific reasoning that undermines efforts to determine the roles that vitamin C may play in human health and disease.

As we continue performing vitamin C research in the future, it is worthwhile to periodically review the literature for experimental approaches that may no longer be valid based on contemporary knowledge. The purpose of this article is to closely examine studies where vitamin C research has “failed” due to methodological, experimental, or design flaws and learn from these errors to help improve future studies, rather than to review studies that have found beneficial effects of vitamin C in human health, as has been done previously [1,3,4,5]. In so doing, we will re-evaluate two common models, cultured cells and experimental animals, and highlight aspects of each system that may contribute to erroneous conclusions. In addition, we will evaluate human research, continuing from previous reviews of the subject [1,2], including an examination of the design and execution of randomized controlled trials (RCTs). Finally, we will explore some of the technical aspects of vitamin C research, in order to promote better awareness of sample handling issues and analytical techniques that are critical for the proper interpretation of study outcomes.

## 2. Review of Studies Using Ascorbic Acid

### 2.1. Ascorbic Acid in Human Cell Culture

The conditions found in a typical cell culture environment promote the oxidation and subsequent degradation of ascorbic acid. Therefore, ascorbate is not usually added to cell culture media, as it often leads to the production of deleterious free radicals and reactive oxygen species (ROS). However, such conditions are non-physiological with respect to vitamin C, which is found in all extra- and intracellular, aqueous solutions *in vivo*. The consequences of such “a-scorbic”––or scorbutic––cell culture environment are not fully understood, but it is obvious that any “ascorbate-dependent” enzymatic reactions, the cells’ redox “milieu”, and antioxidant network must be severely impaired. Furthermore, reintroducing vitamin C to such a cell culture system may give rise to additional artifacts. In this section, we examine the factors that lead to ascorbate oxidation in cell culture media, the artifacts related to the absence of ascorbate in cultured cells or its addition under normal cell culture conditions, and methods that are currently being developed to allow safe addition of ascorbate to cells for physiologically relevant research. Overall, studying vitamin C in cell culture is fraught with many pitfalls and results need to be approached and interpreted with care.

#### 2.1.1. Ascorbate Stability in Cell Culture

The primary concern with the use of ascorbic acid in cell culture is the stability of the molecule under typical incubation conditions. Cell culture incubators use approximately 90%–95% air and 5%–10% CO_2_, resulting in oxygen levels approximately 10–100 times greater than those found in the circulation and in tissues. Increased oxygen tension can promote a pro-oxidant environment in cell culture media [6], possibly generating a wide variety of ROS that can react with, and hence deplete, ascorbate [7]. However, high oxygen levels alone are not sufficient to cause substantial ascorbic acid oxidation. Although the reduction potential of the reduced form of vitamin C, the ascorbate mono-anion (AscH^−^), is sufficient to reduce molecular oxygen to superoxide radicals (Equation 1), the reaction kinetics make this process very slow (estimated second-order rate constant, k_2_ ≈ 10^−4^ M^−1^ s^−1^) [8].

AscH^−^ + O_2_→Asc^•−^ + O_2_^•−^ (1)

**Figure 1:**
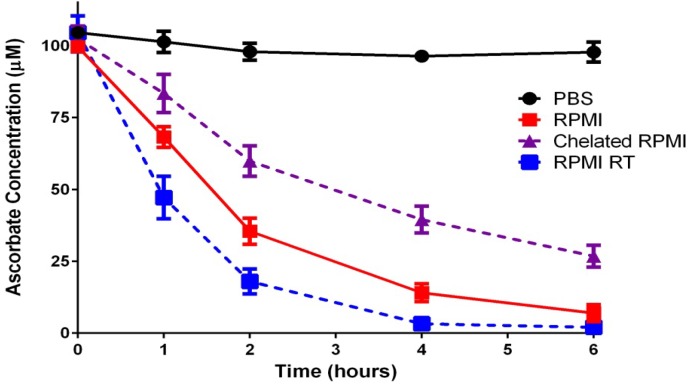
Ascorbate oxidation in buffer or cell culture medium. Ascorbate (100 μM) was added to RPMI 1640 or phosphate-buffered saline (PBS) and monitored over time. Chelated RPMI was used after overnight treatment with Chelex 100 resin and the addition of diethylenetriaminepentaacetic acid (DTPA, 1 mM) as described in Methods. RPMI RT represents media not incubated under 5% CO_2_ but under ambient air at room temperature.

The stability of the ascorbate mono-anion is apparent in deionized water or simple salt solutions: ascorbate added to phosphate-buffered saline (PBS) shows minimal oxidation over a six-h time period in a cell culture incubator (Figure 1, PBS) or when exposed to ambient air at room temperature (data not shown). In contrast, ascorbate incubated in cell culture media under standard conditions is rapidly oxidized [9,10] (Figure 1, RPMI). The composition of the cell culture media plays an important role in the rate of ascorbate oxidation: in serum-free RPMI medium the half-life of ascorbate is about 1.5 h (Figure 1); however, a more rapid loss of ascorbate has been noted in other cell culture media formulations such as MEM or Williams E media (data not shown), including more complex solutions containing serum [6,11,12].

**Scheme 1:**
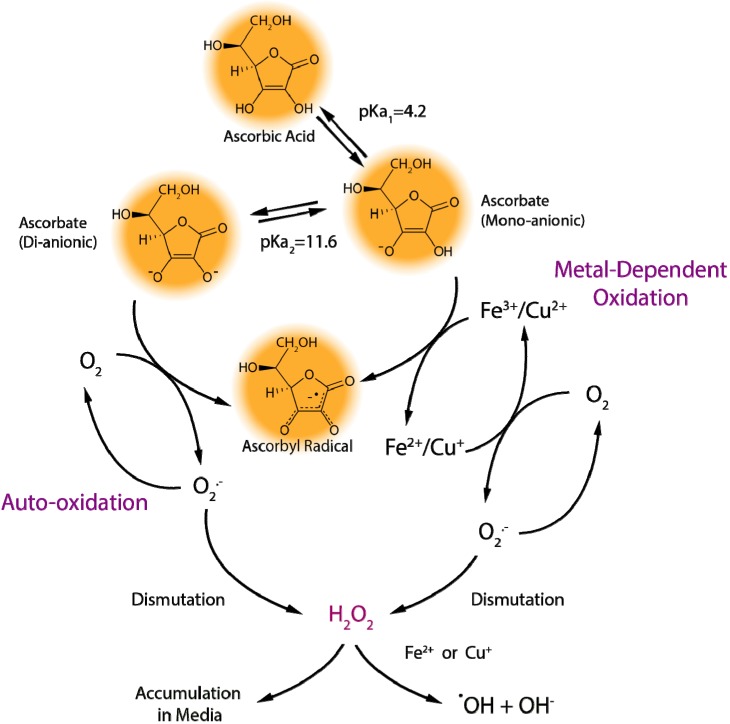
Metal-dependent and metal-independent production of reactive oxygen species by ascorbate in cell culture media.

Iron and copper are present in cell culture media, either added as part of the media formulation or appearing fortuitously, as they are required for normal cell growth and function [13,14]. However, the reduced forms of iron (ferrous iron, Fe^2+^) and copper (cuprous copper, Cu^+^) are able to reduce molecular oxygen to superoxide and, hence, participate in the production of ROS in cell culture systems. The Haber-Weiss reaction (also known as the superoxide-driven Fenton reaction) involves the reduction of the oxidized forms of iron (ferric iron, Fe^3+^) or copper (cupric copper, Cu^2+^) by superoxide (Equation 2) and the subsequent conversion of hydrogen peroxide (formed, e.g., by dismutation of superoxide radicals) to hydroxyl radicals and hydroxide by the reduced metal ions (Fenton reaction, Equation 3) (Scheme 1). Hence, the metal ions act as catalysts and are required only in trace amounts for the Haber-Weiss reaction (Equation 4).

One of the most important biological functions of ascorbate is the reduction of Fe^3+^ or Cu^2+^ in the active site of enzymes, providing electrons used either in the hydroxylation of the enzymes’ substrates or the maintenance of the active-site metal ion in the reduced state [3]. In the case of enzyme-bound metals, the transfer of electrons occurs in a controlled manner, which minimizes deleterious side reactions. However, in a solution of non-protein bound iron or copper, added ascorbate will reduce these metal ions (Equation 5); replacing superoxide in Equation 2, leading to superoxide production and facilitating the flow of electrons into the Haber-Weiss reaction (Equation 4). Even if media formulations are carefully controlled, trace amounts of these transition metals may be found on cell culture glassware, plastic dishes, and reagents, fueling ascorbate oxidation and ROS production [8,15].

Fe^3+^/Cu^2+^ + O_2_^•−^→Fe^2+^/Cu^+^ + O_2_ (2)

Fe^2+^/Cu^+^ + H_2_O_2_→Fe^3+^/Cu^2+^ + OH^•^ + OH^−^ (3)

Fe/Cu

Sum: O_2_^•−^ + H_2_O_2_→O_2_ + OH^•^ + OH^−^ (4)

AscH^−^ + Fe^3+^/Cu^2+^→Asc^•−^ + Fe^2+^/Cu^+^ + H^+^ (5)

In biological fluids and inside cells *in vivo*, a combination of low oxygen levels and protein-bound metal ions greatly reduce ascorbate-mediated pro-oxidant effects [16,17,18]. Consequently, ascorbate in human plasma does not get readily oxidized [19]. By contrast, ascorbate addition to cell culture media results in the production of ROS, including superoxide radicals, hydrogen peroxide (from the dismutation of superoxide radicals), and hydroxyl radicals (Scheme 1) [6,15]. For this reason, ascorbic acid is often mistaken for inducing a pro-oxidant environment in cell culture systems [17,20], although the effects of hydrogen peroxide production specifically may be masked by other media components, such as serum proteins, pyruvate, or α-ketoglutarate [6,11,21,22]. A more accurate description may be that ascorbate unmasks the presence of catalytic transition metals in the cell culture environment [15]. Treating cell culture media with metal chelating agents slows the rate of ascorbate oxidation (Figure 1, Chelated RPMI), increasing the half-life from approximately 1.5 h in standard media (RPMI) to about 2.7 h. Interestingly, metal chelation does not completely prevent ascorbate oxidation (Figure 1), suggesting that other media components also contribute to ascorbate loss [6].

One such contributing factor may be ascorbate auto-oxidation: the direct reaction of ascorbate with molecular oxygen [8]. As stated above, the direct interaction between the ascorbate mono-anion and oxygen is highly unfavorable; however, the ascorbate di-anion (Asc^2−^) rapidly reacts with oxygen (Equation 6). At pH 7.0, the concentration of Asc^2−^ is low, but its contribution to the rate of ascorbate oxidation can become considerable under conditions when pH rises or with increasing concentrations of added ascorbate (Scheme 1) [23]. Cell culture media pH is often stabilized by the addition of sodium bicarbonate to offset the acidic effects of the high CO_2_ environment. However, when this media is exposed to air outside the incubator, the pH value can rise rapidly to 8.0 or higher [11]. RPMI media equilibrated to ambient air accelerates ascorbate oxidation when compared to media in a cell culture incubator (Figure 1, RPMI RT *versus* RPMI), decreasing the half-life from about 1.5 h to less than one hour. As the Asc^2−^ present in solution reacts with oxygen to form dehydroascorbic acid, additional Asc^2−^ ions are generated to re-establish the equilibrium driven by the pH (Scheme 1 and Equation 7). This continuous production of Asc^2−^ would fuel auto-oxidation, which is prevalent when supraphysiological levels of ascorbate are added to cell culture media, since the Asc^2−^ concentration would increase proportionally and greatly enhance oxidation effects. Although concentrated ascorbic acid will lower the pH of cell culture media, investigators usually offset this by the addition of sodium hydroxide to limit the impact of the acidic pH on cells. However, any shift toward a neutral or slightly alkaline environment will increase the metal-independent, pH-driven pro-oxidant effects of ascorbate, which may explain the limited effect of metal chelators to reduce the cytotoxicity of millimolar concentrations of ascorbate towards cancer cells [24].

Asc^2−^ + O_2_→Asc^•−^ + O_2_^•−^ (6)

AscH_2_↔AscH^−^ + H^+^↔Asc^2−^ + 2H^+^ (7)

Ascorbate levels can be maintained in cell culture media by frequent addition of vitamin C [9], but the persistent oxidation will continuously generate dehydroascorbic acid [25] and breakdown products, such as oxalate and threonate [26]. Extracellular concentrations of dehydroascorbic acid in excess of 1–2 μM are considered non-physiological, given its short half-life [25] and rapid uptake and by cells [27,28,29]. Although cells can reduce the dehydroascorbic acid to ascorbic acid, it is currently unclear what effects constant exposure of cells to high levels of dehydroascorbic acid or its breakdown products may have. For example, dehydroascorbic acid exposure has induced stress signaling and cytotoxicity in some cell types, probably due to the loss of NADPH or glutathione needed for dehydroascorbic acid reduction [30]. Oxalate has been shown to exert cytotoxic effects [31], and threonate can impact cell signaling pathways [32]. If anything, these exposures are more likely to generate artifacts as a consequence of the cell culture environment.

#### 2.1.2. “Cellular Scurvy”

Even under conditions of severe vitamin C deficiency, *i.e.*, scurvy, some ascorbate is still present in cells and tissues of humans *in vivo*. As discussed above, cell culture media are not usually supplemented with ascorbic acid, due to its inherent instability in these media. As a consequence, many researchers have reported that cells in culture are devoid of any detectible amounts of ascorbate, even with the use of extremely sensitive HPLC techniques [24,33,34,35,36,37,38]. Similarly, many complete cell culture media containing fetal bovine serum (FBS) have no detectable amounts of ascorbate [33,34,36,38], and our own analysis of various commercial sources of media and FBS has shown identical results (unpublished observations). The effects of these ascorbate-free conditions are not well defined or understood. However, it should be recognized that many immortalized cell lines likely have been maintained under scorbutic conditions for generations. In this manner, a whole host of cell culture artifacts may be expected when ascorbate is reintroduced into the system.

Cells in culture can be maintained without ascorbic acid because it is not essential to cell growth and division. The biological functions of ascorbate as an electron donor in enzymatic synthesis pathways do not have an absolute requirement for ascorbate [3,39,40]. These enzymes can use other reducing substrates as sources of electrons [39,41], and enzyme activity can still occur in the absence of ascorbate, albeit at a far decreased rate [42]. In particular, the α-ketoglutarate-dependent dioxygenases, such as those involved in collagen synthesis and regulation of hypoxia-inducible factor 1α (HIF-1α), do not require ascorbate as part of the normal catalytic cycle; ascorbate is only needed to rescue the enzyme should an uncoupled enzymatic reaction occur [3]. It has also been suggested that ascorbate may function to maintain intracellular iron in the ferrous state, making it available to replenish or replace ferric iron in the active site of these enzymes [43]. It is possible that cells in culture adapt by increasing ferrous iron uptake and turn-over of iron-containing proteins, partially circumventing the need for ascorbic acid. Regardless of the mechanism, it is evident from cell culture studies that “ascorbate-requiring” enzymes, such as those involved in collagen synthesis [44], degradation of HIF-1α [43], norepinephrine and α-amidated peptide synthesis [45], and histone and DNA demethylase activity [46], still exhibit some residual activity in the absence of ascorbate. However, these enzymes have diverse effects in different tissues, and their activity in an ascorbate-free environment may not be reflective of their roles *in vivo*.

On the other hand, normal physiological functioning of cells can be recapitulated when ascorbate is provided. Ascorbate appears to play an important role in the normal function of cultured endothelial cells, raising antioxidant protection, reducing oxidative stress and damage, and increasing eNOS activity when compared to cells devoid of vitamin C [33,38]. These effects on eNOS, at least, appear dependent on the ability of ascorbate to enhance the stability of tetrahydrobiopterin [34] and influence AMP-activated kinase (AMPK) activity [47]. In addition, ascorbate supplementation of cultured endothelial cells tightens cell-to-cell junctions that are critical for maintaining an endothelial barrier *in vivo* [48] and regulates NADPH oxidase activity [49], a critical component of the inflammatory response.

It is important to note that the effect of ascorbate supplementation may also greatly vary by cell type. Many of the aforementioned effects of ascorbate are observed in primary cell lines. Although propagated in the absence of ascorbate, the response to ascorbate supplementation in these cells reflects responses seen *in vivo*. Cancer cell lines and other immortalized cells, however, often show cytotoxic effects in response to ascorbate addition that are not observed in primary cell lines [24]. This may be the result of adaptations that have accumulated in these cells due to the “culture shock” that alters the normal physiological responses to stimuli [6], possibly involving iron dysregulation or aberrant cell signaling responses.

#### 2.1.3. Proper Use of Ascorbate in Cell Culture

Cell culture study designs may have a large impact on the results obtained with ascorbic acid. To minimize artifacts, cell culture experiments should replicate *in vivo* conditions as closely as possible. Ascorbate levels in media should be maintained within the physiological range of human plasma (about 5–100 μM), and the use of supraphysiological concentrations should be avoided, unless conditions of intravenous vitamin C infusion are being mimicked [24]. When ascorbic acid is added to cell culture, the loss of ascorbate in the media competes with the intracellular accumulation of ascorbate. Although cells may accumulate ascorbate, once the media is depleted of ascorbic acid, intracellular ascorbate levels decline slowly through oxidation or efflux [33,34,35,38], once again returning cells to a depleted state. Meanwhile, degradation products may accumulate in the media or cells, which would normally be removed under physiological conditions. In addition, cells without ascorbic acid are not a proper control for ascorbate treatment, as some level of ascorbate is always present in all cells of the human body.

Ascorbate should be added to culture media in a way that limits the rate of ascorbate oxidation and the effects of ROS that may be formed. The use of serum-free media that has been supplemented with transferrin to control iron or copper redox chemistry shows great promise in stem cell studies [46]. The stability of ascorbate can be enhanced by low oxygen growth conditions and the use of stabilized derivatives of ascorbate such as ascorbate-2-phosphate (AAP) that cannot participate in redox chemistry outside the cell yet can maintain physiological intracellular ascorbate levels [9]. Furthermore, pyruvate or α-ketoglutarate in cell culture media can be used to blunt the effects of any hydrogen peroxide formed [21,22], although they will not prevent the loss of ascorbic acid.

Due to the inherent instability of the molecule, there is an absolute necessity for monitoring ascorbic acid levels in media and cells during cell culture experiments. As with animal and human studies described below, this is the only method currently available to assess the vitamin C status of cells, and is a valuable tool for understanding the mechanisms of ascorbate’s biological actions. Unfortunately, ascorbate levels are rarely measured in cell culture, animal, or human studies, which severely limits their validity and any conclusions that can be drawn.

Despite precautions, conventional cell culture conditions will promote an environment in which ascorbate artifacts are commonplace. Culturing cells with vitamin C requires control over many aspects of the media and culture conditions that has heretofore been lacking. Monitoring ascorbate levels and limiting oxidation may not be sufficient to fully recapitulate the physiological roles of vitamin C. While redesigning cell culture systems to support biologically relevant reactions of ascorbic acid and eliminate artifacts may limit the practicality of experimental designs, these changes are necessary for cell culture models to have continued use in vitamin C research.

### 2.2. Animal Studies Involving Ascorbic Acid

Since it can be synthesized *de novo* and is not an essential nutrient for most animals, ascorbate should not be referred to as a vitamin in these animals. Conceptually, this questions the use of most experimental animal models, in particular rats and mice, to study the role of ascorbic acid in human health and disease [50]. This echoes recent studies that have cast doubt on the pervasive use of rodent models to mimic human inflammatory responses [51]. Although genetically variant strains incapable of synthesizing ascorbic acid have been established, none of these models fully recapitulate vitamin C transport functions or the effects of vitamin C depletion and repletion observed in humans or guinea pigs [50]. Indeed, there is evidence that regulation of vitamin C transport was altered during the evolutionary loss of l-gulonolactone oxidase (GULO) expression, which does not occur in genetic knockouts. Here, we examine the current issues related to the use of animal models in ascorbate research. Overall, if animals are to be used, the limitations imposed by the model should be well understood. Animal experiments should be avoided if comparable studies can be performed in human subjects.

#### 2.2.1. When a Vitamin in not a Vitamin

Most animal species express a functional copy of l-gulonolactone oxidase, an enzyme with the synthesis of l-ascorbic acid as its only known function. In these animals, the regulation of GULO activity appears to depend primarily on substrate availability, namely the production of gulonolactone as a branch product from glucuronate synthesis [26] derived from UDP-glucose, a product of glycogen breakdown [52,53]. Thus, agents that stimulate glycogenolysis also stimulate ascorbate synthesis when an animal is in a fed state (*i.e.*, when glycogen is present); in contrast, prolonged fasting causes ascorbate synthesis to decline [54]. UDP-glucuronate is also needed for glucuronidation of xenobiotics, and there is a correlation between ascorbate synthesis activity and xenobiotic metabolism [26].

In each of the few mammalian species that do not synthesize ascorbic acid, such as guinea pigs, fruit bats, and primates, the loss of GULO has occurred at a genetic level. Although the mutations accumulated in this gene differ [55], the result is essentially the same: a loss of GULO activity. These species have adapted to the loss of *de novo* synthesis by consuming primarily plant sources of ascorbate. While all non-synthesizing animal species are at risk of developing scurvy and may die when vitamin C intake ceases for an extended period of time, this is a condition that does not occur normally in other animals, including most commonly used experimental animal models, such as mice and rats. Thus, in contrast to ascorbic acid-synthesizing species, in non-synthesizing species, including humans, vitamin C absorption is required to prevent deficiency and maintain health. This represents a fundamental shift from a perspective of diet-health interactions, as the absorption-derived *versus* glycogen-derived source of ascorbic acid may represent differences in ascorbate transport and carbohydrate metabolism that exist in humans and synthesizing animals.

Although evidence in the literature is limited, both rats and mice appear to poorly absorb ascorbate from the diet. Early studies on the small intestine in rats revealed that ascorbate uptake is a passive process, resulting mainly in intestinal mucosa accumulation but not transport to the circulation [56]. Several studies have demonstrated a profound difference in absorption between the rat and guinea pig small intestine [57,58,59], the latter displaying a robust transepithelial transport system that is sodium-dependent [59,60,61], similar to the human ileum [61,62]. A recent study in rats monitored the absorption of a single oral dose of ascorbic acid or dehydroascorbic acid given by gavage. The administration of 12 mg of dehydroascorbic acid led to a significant increase in plasma ascorbic acid concentration, but administration of 12 mg of ascorbic acid did not [63]. An inability to efficiently transport vitamin C is also seen in feeding studies, where mice fully capable of synthesizing ascorbate required at least 45 mg of ascorbate per day in their diet to show any significant increases in plasma ascorbate concentration [64]. Although it is difficult to extrapolate these doses to humans, allometric scaling based on calorie consumption suggests that a dose of 45 mg in a 20-g mouse is equivalent to about 3 g in a 70-kg person [65].

Studies on the bioavailability of different forms of ascorbic acid supplements also support the notion that ascorbate is poorly absorbed by rodent models. For example, Ester-C, a calcium ascorbate-threonate mixture, is reported to be more bioavailable in ODS rats than an equivalent dose of ascorbic acid [66]. However, the same comparison of supplements shows that Ester-C has a lower rate of absorption—and certainly no enhanced bioavailability—in human volunteers [67]. Furthermore, work in GULO knockout mice suggested an enhanced bioavailability of vitamin C contained in a kiwifruit puree compared to a pure ascorbic acid supplement gel [68]. However, more recent human data suggest that vitamin C from kiwifruit or a supplement is equally bioavailable [69].

Overall, these data support a species-specific route of ascorbate absorption. While indirect absorption of ascorbate may occur in rats and mice—likely mediated by ascorbic acid oxidation and transient formation and transport of dehydroascorbic acid—there is clear evidence of an active, sodium-dependent transport of vitamin C in guinea pigs and humans. Therefore, the use of rats or mice as a model of human vitamin C absorption and metabolism is ill advised. As indicated above, such studies with experimental animals should be avoided if they can be performed in humans, unless the purpose of the study is to better understand ascorbic acid absorption and metabolism in rodents. More importantly, no conclusions should and can be drawn from such studies for human vitamin C transport or metabolism.

Poor uptake of dietary ascorbate, or complete lack of it, is expected in animals that synthesize ascorbate, as they do not have a need for dietary ascorbate. In addition, high levels of ascorbate in the intestine would likely cause down-regulation of tissue ascorbate synthesis. However, the relationship between absorption and synthesis does not appear to be this simple. Genetically altered rat and mouse models lacking ascorbate synthesis have low tissue levels of ascorbate without supplementation [64,68,70,71]. Although these animals absorb dietary ascorbate, the levels needed to prevent scurvy or saturate tissues are relatively high compared to guinea pigs and humans on a body-weight basis (Table 1). These levels are especially high when contrasted to food sources of ascorbate. As an example, to saturate all tissues, GULO knockout mice need to be supplemented with 3.3 g/L of ascorbate in the drinking water, resulting in an intake of approximately 16.5 mg per day [68,72]. Based on allometric scaling, this dose in mice corresponds to about 1 g per day in a 70-kg person [65]. Although the metabolic rate of these animals likely contributes to high ascorbate requirements, this does not sufficiently explain data supporting an excessive inefficiency of intestinal absorption.

#### 2.2.2. Transporter Troubles

Based on the evidence presented above, animals unable to synthesize vitamin C appear to display an active transport system in the intestine that is both sensitive to varying levels of ascorbic acid and sodium-dependent. This implicates a role of the sodium-dependent vitamin C transport (SVCT) proteins. SVCT1 has been implicated in dietary ascorbate absorption in human enterocytes, as it is found primarily on the luminal side of intestinal cells and involved in trans-epithelial ascorbate transport [73,74,75]. However, mice genetically modified to remove functional expression of the SVCT1 gene (Slc23a1^−/−^) show similar intestinal ascorbate absorption as wild type mice [76], suggesting SVCT1 is not involved in this process in these animals. Indeed, the concentration of ascorbic acid needed in the drinking water of GULO knockout mice to maintain tissue saturation (3.3g/L or 18.75 mM) far exceeds the transport capacity of SVCT1, which has a measured *K*_m_ below 250 μM [77]. On the other hand, the ability of rats to absorb dehydroascorbic acid has been linked to the intestinal expression of glucose transport proteins (GLUTs), namely GLUT2 and GLUT8 that show K_m_ values of approximately 2–3 mM [63].

**Table 1:** Animal models for vitamin C research and comparison to humans.

Species/Strain	GULO Status	Maintenance Dose (mg/kg Body Weight) *	Saturation Dose (mg/kg Body Weight)	Comparisons to Humans and Other Models
Mouse (wild-type)	Functional	n/a	n/a	Expresses GLUT4 on erythrocytes [78]
*Sfx* Mouse	Complete deletion	~20	>100	Spontaneous mutant model that develops spontaneous bone fractures and bone fragility not seen in other GULO knockout mice [71,79]
GULO^−/−^ Mouse	Exon 3 & 4 deleted	~20	~600	Genetically engineered mouse model that displays blood vessel fragility [80] Possible muscle weakness independent of vitamin C status [81]
SMP30^−/−^ mouse	Functional	~20	~240	Not a GULO knockout [82]; may have residual synthesis activity Multiple aging effects independent of vitamin C status [50]
Rat (wild-type)	Functional	n/a	n/a	Expresses GLUT4 on erythrocytes [78] Preferential absorption of dehydroascorbic acid in gut [63] Lack of active ascorbate transport in intestine [49,50,51]
ODS Rat	GULO point mutation	~10	~200	Spontaneous mutant model that develops hind limb bone disorders [83] Vitamin C deficiency lowers blood pressure [84] Vitamin C deficiency protects from ischemic injury [85] GULO mRNA and protein still expressed [86]
Guinea Pig	Multiple exons lost	~2	~27	GULO gene deletion during evolution [55] Expresses GLUT1 on erythrocytes, similar to humans [78] Active ascorbate absorption in gut, similar to humans [49,50,51] Vitamin C deficiency exaggerates cardiovascular decline with age, similar to humans [87,88,89] Other similarities to human vitamin C supplementation [50]
Human	Multiple exons lost	~0.15	~3	

n/a: not applicable or unable to determine due to endogenous ascorbate synthesis; * Approximate dose needed to prevent symptoms of scurvy.

Unfortunately, little is known about differences in expression or regulation of the SVCTs between humans, guinea pigs, rats, and mice. Comparison of the amino acid sequences of mouse, rat, and human SVCT1 shows that seven amino acid residues are missing from the human SVCT1 sequence [90]. This deletion creates a potential protein kinase C (PKC)-binding site in the human sequence not found in rats or mice. Stimulation of PKC has been implicated in membrane trafficking of human SVCT1 [91]. Additionally, the *C*-terminal sequence of rat and mouse SVCT1 has a one-amino acid change in a critical four-amino acid sequence required for apical targeting of the transport protein [92]. SVCT2, responsible for uptake of ascorbate from the blood stream, contains an additional 56 amino acids in the *N*-terminal region of the human protein sequence that are not found in rats and mice [90]. The only study to date on a species difference in SVCT regulation found that buthionine sulfoximine (BSO), a glutathione synthesis inhibitor, reduced the expression of both SVCT1 and SVCT2 in rat liver cell lines, a phenomenon not observed in human hepatoma cells [93].

Although the absorption of dehydroascorbic acid is not considered a major pathway for the maintenance of whole body ascorbic acid levels, it is considered an important scavenger pathway to maintain cellular ascorbate levels if extracellular ascorbate is oxidized to dehydroascorbic acid [94]. Since human erythrocytes do not express SVCT proteins [95], ascorbic acid transport across the plasma membrane is facilitated by dehydroascorbic acid uptake mediated through GLUTs.

Recent evidence suggests that GLUT1 is responsible for dehydroascorbic acid uptake in human red blood cells, enhanced by the co-expression of a protein called stomatin during erythropoiesis [78]. By contrast, mice lose GLUT1 during maturation, and GLUT4 is the predominant glucose transporter expressed in adult mouse erythrocytes [96]. GLUT4, by contrast, has diminished capacity to transport dehydroascorbic acid [97], which is reflected in the transport capacity of murine erythrocytes [78]. Interestingly, GLUT1 expression and the associated dehydroascorbic acid transport in red blood cells are found only in species unable to synthesize ascorbate—lacking from every animal with endogenous ascorbate-synthesis capacity, even in closely related species such as chinchilla and guinea pigs, or lemurs and margot monkeys [78]. Furthermore, this remarkable switch in glucose transport proteins may be indicative of differences in global gene expression patterns between ascorbate synthesizing *versus* non-synthesizing animals.

#### 2.2.3. Choose Models with Care

Although wild-type rat and mice models are still being employed for ascorbate research, the presence of endogenous synthesis alone would suggest that these animals represent a poor model for understanding the role of vitamin C in human health and disease. At the very least, animal models without endogenous ascorbate synthesis should be used. Five rodent models exist, although each with their own limitations (Table 1). Only one of these models, the GULO knockout mouse, was specifically engineered to disable ascorbic acid synthesis [80]. The other mouse and rat models are not genetically engineered and display individual characteristics that poorly recapitulate the effects of human vitamin C deficiency [50]. On the other hand, guinea pigs have lost GULO activity during evolution and likely display compensatory genetic adaptations similar to humans relating to the loss of ascorbate synthesis, such as GLUT1 activity on erythrocytes (see above). Although guinea pigs are currently being employed by some research groups, the lack of molecular and genetic tools for this animal model will likely drive the continued use of rats and mice models instead.

Regardless of the species, experimental conditions, or route of ascorbate administration, it needs to be stressed that measuring ascorbate levels is absolutely necessary for any animal experiment. One of the most frequent assumptions is that dietary ascorbate will always result in a change in tissue and plasma ascorbate levels. However, the relative contributions of *de novo* ascorbate synthesis (if present), absorption of dietary ascorbate, and ascorbate distribution throughout the body are not inherently predictable and must be directly measured. Studies using oral ascorbate administration can be complicated by animals with poor absorptive capacity, and these animals may rely on the production of dehydroascorbic acid in the intestine. Furthermore, stable ascorbate derivatives used in animal diets, such as ascorbate phosphate or ascorbate palmitate, are not well studied and may not distribute throughout the body as ascorbate would if provided by itself. It is also not recommended to rely on intravenous, intraperitoneal, or subcutaneous ascorbate injections, since they may cause the production of hydrogen peroxide in the extracellular space [98].

Since ascorbate is a dietary factor in humans *versus* a product of carbohydrate metabolism in mice and rats, and there are differences in SVCT and GLUT regulation between species, the results of most animal studies with ascorbic acid cannot be extrapolated to humans. Continued evaluation of rodents as relevant models to study ascorbic acid in human health and disease depends on a thorough understanding of the differences between rodents and humans with respect to ascorbate metabolism, regulation, and biological functions. However, it seems apparent that, aside from the guinea pig, animal studies should be avoided as much as possible, with continued focus placed on conducting relevant human studies instead.

### 2.3. Human Studies with Vitamin C

Randomized controlled trials are considered the “gold standard” by the Institute of Medicine’s Food and Nutrition Board for determining efficacy of micronutrients, including vitamin C, in promoting human health and preventing or treating disease [1,2]. However, RCT study designs have serious limitations and pitfalls, and require careful scrutiny to avoid misinterpretation of results and erroneous conclusions. The drive to establish correlations in prospective cohort studies or show a treatment effect in RCTs often ignores assessment of intermediary biomarkers or other biological measures that could provide insights into mechanisms and help establish causation. Indeed, many vitamin C supplementation studies are performed in combination with other supplements, most often “antioxidant vitamins” E and β-carotene, and fail to assess the subjects’ vitamin C status at baseline and following supplementation or biomarkers related to vitamin C’s proposed mechanism of action, e.g., antioxidant or anti-inflammatory effects [2]. Understanding the study population, limitations of study design, and the nuances of the ascorbic acid chemistry is necessary to avoid many of these pitfalls and artifacts. Furthermore, myths about the health effects of vitamin C supplementation plague the entire body of research and may prompt the spread of misinformation. In this section, we explore the limitations to human research with vitamin C and review the myths surrounding vitamin C supplementation. In the future, it will be necessary to promote a new approach to conducting vitamin C research in humans.

#### 2.3.1. Vitamin C RCTs: Failures in Design

Many, but not all, prospective cohort studies have observed inverse associations between vitamin C intake or plasma levels and the incidence of chronic diseases, including coronary heart disease, ischemic stroke, hypertension, and certain types of cancer [1,15]. However, several large RCTs have shown no benefit of vitamin C supplementation when taken alone or in combination with other micronutrients [28,51]. This apparent failure of vitamin C supplements to affect human health can be attributed to many factors related to study design. The most predominant is the use of the standard RCT study design, which is intended to test the safety and efficacy of a pharmaceutical drug in individuals that are at high risk or are suffering from a condition or illness. By contrast, enrollees in vitamin C supplementation studies, and diet-related RCTs in general, are usually health-conscious individuals who are likely to consume an above-average diet and maintain a healthy body weight [99]. As a consequence, these individuals have a lower disease incidence and a better nutritional status, including vitamin C, than the general population—both of which negatively affect the statistical power of the study. Statistical power is further compromised by the fact that there is no true placebo group in these studies, as even the non-supplemented subjects continue to obtain vitamin C from their diet throughout the duration. These and other serious flaws in study design, including lack of a single supplement (vitamin C only), quality of the methodology employed, and lack of discrimination by genetic polymorphisms, have led some to the unfortunate conclusion that very few well-designed, well-controlled trials of supplemental vitamin C have ever been conducted [2].

One reason previous studies have failed to show health benefits of vitamin C may be the assumption that an individual’s plasma or body ascorbate status directly reflects their dietary or supplemental intake of vitamin C. To the contrary, analysis of food frequency questionnaires has revealed that there is little correlation between assessed vitamin C intake and plasma ascorbate levels [100], likely due to inaccuracies in dietary assessment methodology using food frequency questionnaires or food diaries, inaccuracies in the USDA nutrient database, loss of ascorbate during storage, cooking or processing, and large inter-individual differences in vitamin C absorption and metabolism. An example of the latter is the lower plasma ascorbate levels observed in the elderly when compared to younger adults consuming equivalent amounts of vitamin C [101], suggesting changes in absorptive capacity with age. In addition, smoking, chronic aspirin use, high alcohol consumption, high BMI, and low socioeconomic status [102] are all factors that have been associated with lower plasma vitamin C levels. Furthermore, genetic variation in SVCTs, haptoglobin, and glutathione *S*-transferases also may lead to altered plasma ascorbate levels depending on the various single nucleotide polymorphisms involved [103]. In each of these cases, the exact relationship of plasma vitamin C status with vitamin C consumption is unclear. However, this explains why food frequency questionnaires have little predictive value for evaluating the effect vitamin C consumption on disease risk, while plasma ascorbate levels display clear inverse relationship [104]. Therefore, the use of dietary analysis in studies pertaining to vitamin C should be only a secondary measure of vitamin C status, at best. The gold standard must be measurement of plasma ascorbate levels.

Human pharmacokinetic data show that there is a sigmoidal dose-response relationship between plasma ascorbate levels and vitamin C dose for both men [105] and women [106]. Those with frank deficiency have plasma ascorbate values below 11 μM and are at risk for scurvy because corresponding tissue levels are low. Marginal deficiency (<23 μM) and suboptimal concentrations (<50 μM) are levels that exist on the steep part of the dose-response curve, thought to be indicative of increasing levels of ascorbate in most tissues, based on correlations with ascorbate levels in circulating leukocytes. Plasma concentrations start leveling off at doses above 200 mg/day and approach maximal levels in the range of 60–90 μM, when the threshold levels for renal reabsorption are reached and leukocytes also are saturated with vitamin C.

There are limitations to these pharmacokinetic data that must be recognized. First, the studies were performed in a small number of young, healthy individuals and, hence, are limited in their statistical power. As described above, many factors can influence the relationship between plasma and dietary ascorbate, including age and disease status, which may affect vitamin C transport and metabolism. Therefore, vitamin C pharmacokinetics may be substantially different in old or diseased individuals compared to young, healthy subjects. Second, we cannot assume that tissue saturation occurs in every organ along the same continuum of plasma ascorbate levels. Studies in animals show preferential uptake and retention of ascorbate in organs that have high requirements for the vitamin [68,70,107]. Thus, the brain may saturate at lower ascorbate intake and plasma levels than other organs, such as liver or circulating cells. Data in human volunteers suggest that in skeletal muscle ascorbate is more responsive to changes in plasma ascorbate status than in neutrophils or mononuclear cells [108], suggesting different routes of vitamin C transport and levels of tissue saturation. One study showed a continued uptake and no apparent saturation of ascorbate in the human eye lens with increasing plasma ascorbate levels [109]. Thus, the implication here is that transport rate and saturation point in various cells and tissues of the body are variable and may not be directly extrapolated from plasma ascorbate levels.

From the above considerations, three critical issues emerge in relation to RCT design. First, individuals recruited for a research study should have low plasma ascorbate levels at baseline to increase the likelihood of affecting changes in ascorbate status in tissues through vitamin C supplementation. Subjects already consuming enough vitamin C to provide near-maximal or saturating plasma and tissue levels of ascorbate are highly unlikely to demonstrate any further biological or health effects upon vitamin C supplementation. Second, the intervention must be proven effective, demonstrating—at the very least—an elevation in plasma ascorbate steady-state levels. Again, if a research subject’s vitamin C status does not change, no changes in health or disease outcomes can be expected unless it can be supported by an alternate mechanism. In many cases, no biological effect can be expected of increasing vitamin C levels if no functional deficit is present. For instance, although studies support the use of vitamin C in improving vascular function and reducing blood pressure [5], continued supplementation of vitamin C when plasma levels are already at saturation will not yield additional vasodilation, and changes in blood pressure are not expected if a subject is already within a healthy blood pressure range. Lastly, in the absence of tissue ascorbate measurements, the study design and endpoints must relate to our knowledge about the distribution of vitamin C in the body. For example, if brain ascorbate levels are near saturation at low vitamin C intake and plasma levels, it is unreasonable to expect an effect on brain function over a wide range of intake and plasma ascorbate levels.

Along with attention to these issues, there needs to be a push toward measurement of mechanism-based endpoints and clinically-relevant, intermediary biomarkers to assess the effects of vitamin C supplementation. As mentioned above, in biological systems vitamin C always acts as a reductant, which may be expressed as antioxidant, anti-inflammatory, enzyme cofactor, or pro-oxidant activity, depending on the specific context. Commonly in clinical trials, vitamin C is assumed to act as an antioxidant or an anti-inflammatory without actual measurements to support such a role, which limits the evaluation of the trial as a successful intervention and the interpretation of the data. Measures of oxidative stress, such as F_2_-isoprostanes [110], serve as clinically relevant markers suggesting an antioxidant effect, while changes in circulating levels of inflammatory markers such as C-reactive protein or soluble cellular adhesion molecules can support an anti-inflammatory effect [111]. Without these corroborating data, results from RCTs will continue to be limited in impact and relevance.

With correct study design, it is generally believed that quality research data can be obtained from human subjects. However, the costs of large, tightly controlled RCTs with vitamin C alone are likely to be prohibitive to the implementation of such studies. Therefore, it is likely that smaller, high-quality intervention trials will have to suffice in the future.

#### 2.3.2. Technical Issues of Human Studies

Vitamin C research is heavily dependent on accurate assessment of ascorbate in biological samples. Unfortunately, there is no standard method for measuring vitamin C that has been applied across the field. It is generally acceptable practice to preserve samples in acid or methanol after sample extraction. Furthermore, it is preferred that a direct measure of ascorbic acid be made, such as HPLC separation followed by electrochemical detection (ECD). Other chromatographic methods are generally avoided, especially those that require oxidation of the sample followed by derivatization as these procedures can generate erroneous results. The detection of dehydroascorbic acid cannot be achieved with ECD unless the sample is first treated with a reductant, obtaining total ascorbate levels from which dehydroascorbic acid levels can be inferred [112,113]. However, the value and interpretation of dehydroascorbic acid measurements in biological samples is questionable (see below).

**Figure 2:**
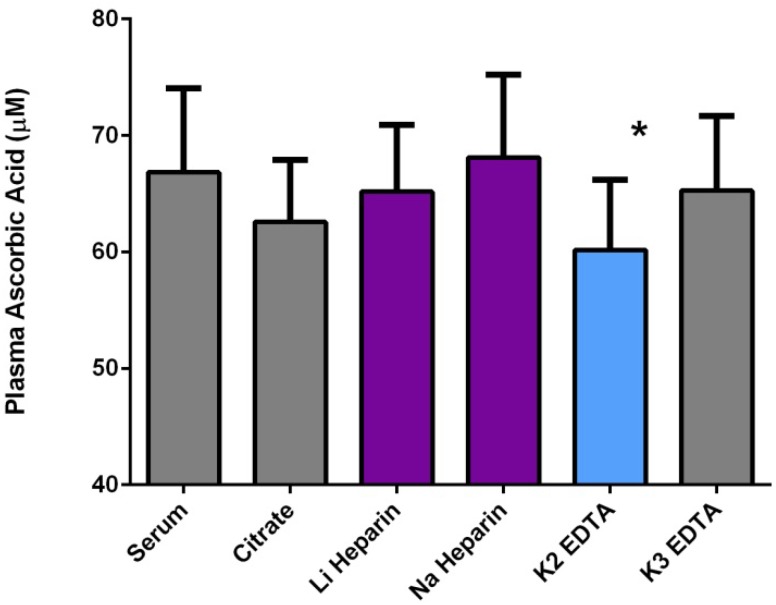
Vacutainer effects on plasma ascorbate levels. Plasma ascorbate concentrations were determined from different anticoagulant-containing or untreated vacutainers as described in Materials and Methods. Plasma ascorbate means are the average and standard error from five different subjects. ANOVA analysis shows a significant (*p* < 0.05) decline in plasma ascorbate levels in K_2_ EDTA vacutainers when compared to lithium or sodium heparin vacutainers as denoted by asterisk (*).

The labile nature of ascorbic acid outside the body underscores the need for controlled conditions during collection, processing, and storage of biological samples. In many human studies, poor standards in obtaining blood or tissues specifically for vitamin C analysis are complicated by a lack of controlled sample handling in many clinical settings. Phlebotomy is common practice, but many factors can contribute to the instability of ascorbic acid in biological samples. Although the use of vacutainers for plasma samples is generally acceptable for vitamin C analysis, the choice of vacutainer type and anticoagulants can influence the results obtained [114]. As an example, plasma ascorbate levels were determined from five individuals using various anticoagulants or no anticoagulant (serum) as a control. Despite individual variability in plasma ascorbate levels reflected in the standard error, the data (Figure 2) suggest that the use of K_2_ EDTA vacutainers results in a significant loss of ascorbate compared to both sodium and lithium heparin containers. Consistent with these data, EDTA has been shown to accelerate the oxidation of ascorbate in whole blood and plasma [115,116] and be unable to prevent the loss of ascorbate in the presence of iron or copper [8]. In addition, it has been suggested that fluoride and serum vacutainers should be avoided for vitamin C analysis [114].

**Figure 3:**
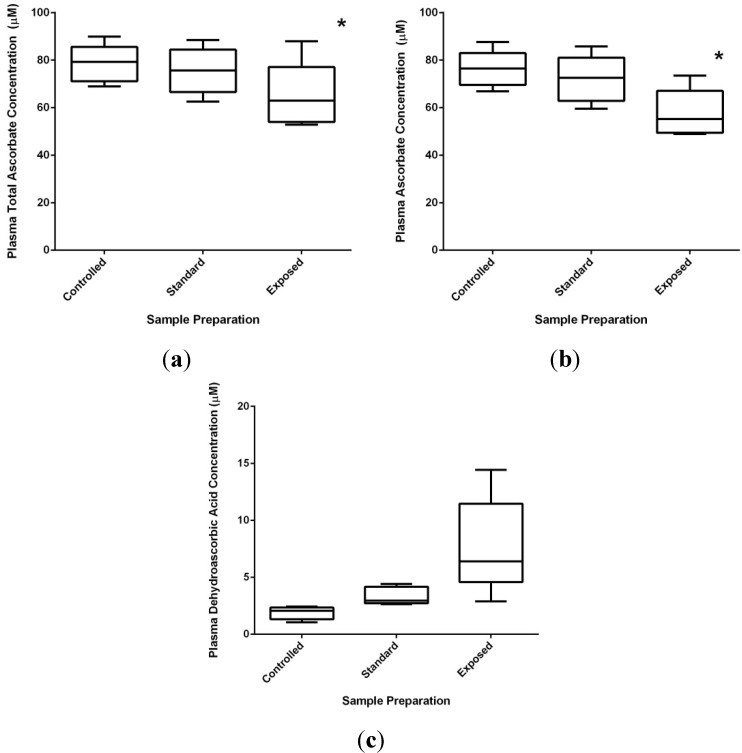
Effects of sample handling on plasma ascorbate and dehydroascorbic acid concentrations. Vacutainers with blood samples were prepared under oxidation controlled, standard and exposed conditions as described in Materials and Methods. Plasma total ascorbate (**a**) and reduced ascorbate (**b**) levels are highest in controlled samples, showing declines under standard and exposed preparations that are reflected in calculated dehydroascorbic acid levels (**c**). Significant changes were observed in the exposed group when compared to controlled or standard samples as determined by ANOVA, and denoted with an asterisk (*).

The oxidation of vitamin C in plasma is accelerated by heat, light, and elevated pH, similar to cell culture media as described above. Sample mishandling can cause the aberrant generation of dehydroascorbic acid in the sample and, over time, will cause a loss of total ascorbate. For instance, by careful preparation of the sample under nitrogen and limited exposure to heat and light, dehydroascorbic acid levels can be minimized (Figure 3). More reasonable, standard preparation methods with brief exposures to air, light, and heat result in little change in plasma ascorbate levels (Figure 3b). On the other hand, exposing samples to room temperature for hours not only can result in a significant decline in (reduced) ascorbic acid, but a loss of total ascorbic acid as well (Figure 3a). Not only does this result in an inaccurate estimate of dehydroascorbic acid levels in the exposed sample (Figure 3c) but the loss of total ascorbate suggests degradation of dehydroascorbic acid has also occurred.

The implication of these data (Figure 3) and others [114,115,116] is that care in sample handling with concern for ascorbate oxidation is crucial for accurate ascorbate analysis. Study designs must incorporate specific handling conditions of samples intended for ascorbate analysis (ideally by immediate plasma isolation, rapid acidification, and freezing below −20 °C) to avoid misinterpretations compounded by the use of poorly preserved samples. Furthermore, it also suggests that the presence of dehydroascorbic acid in clinical samples is more a measure of sample handling than a biologically relevant marker of *in vivo* oxidative stress.

#### 2.3.3. Health Effects of Vitamin C: Reality *versus* Mythology

Many excellent evidence-based reviews have summarized the health effects of vitamin C, focusing on vitamin C’s possible role in the prevention or treatment of cardiovascular disease, cancer, diabetes, and other diseases [1,2,4,117]. However, there are a number of health effects attributed to vitamin C supplementation that are not supported by controlled trials with fundamental, mechanism-based endpoints. Opponents of vitamin C supplementation have sensationalized studies that have suggested deleterious health claims, most of which are of little consequence to the general population. For example, since vitamin C promotes iron absorption [118], this led to claims that vitamin C supplements were detrimental in individuals suffering from iron overload and hemochromatosis. On the contrary, in animals and humans with iron overload, high plasma ascorbate levels are protective against oxidative damage induced by excess iron [18,19]. Indeed, the recommendation for these individuals is to not avoid ascorbate, but instead limit dietary iron intake.

Another commonly cited risk of ascorbate supplementation is the formation of kidney stones, as oxalate is a product of dehydroascorbic acid breakdown. The evidence linking ascorbate supplement use and incidence of kidney stones in otherwise healthy individuals is mixed [119,120,121]. Large amounts of oxalate derived from ascorbate would require excessive vitamin C oxidation, but the mechanism underlying this oxidation has not been explored. Studies that suggest an increased risk observed it in individuals taking more than 1000 mg of vitamin C per day, in far excess of the amount that can be obtained from food sources. Without further study, it is prudent to caution individuals with kidney disease or a history of kidney stones against taking large amounts of vitamin C supplements. Unfortunately, this has also resulted in practitioners to advise individuals undergoing dialysis to severely restrict vitamin C consumption, leading to deficiencies [122].

The consumption of large doses of vitamin C supplements has also been occasionally associated with skin rash, heartburn, nausea, and diarrhea. These are usually the result of the formulation of the vitamin C tablets but may also be caused by excessive consumption of vitamin C in a short period of time. Large doses of vitamin C have been anecdotally associated with vitamin B_12_ deficiency and systemic conditioning (also known as “rebound scurvy”), conditions that have never been documented clinically [123]. Patients with glucose-6-phosphate dehydrogenase (G6PD) deficiency have also been cautioned against taking vitamin C supplements, due to reports of hemolytic anemia that have also not been substantiated [124].

One of the most persistent health claims for vitamin C supplementation is the prevention and treatment of the common cold. Recent meta-analyses of the data from 70 years of placebo-controlled trials demonstrated an overabundance of poorly controlled trials with no apparent focus on the mechanism and biological relevance of vitamin C supplementation [125,126]. Despite the large number of research studies, the evidence supporting the effects of vitamin C supplements on cold incidence and duration has been relatively weak, with the exception of marathon runners, skiers, soldiers in subarctic conditions [125], or individuals with chronic gastritis [127]. A tendency to include older data is a common pitfall for the analysis of vitamin C research, performed when our understanding of vitamin C in biology was limited, and thus allowing for a bias toward poorer designed studies. Our understanding of vitamin C’s role in biology has improved over time, leaving a re-analysis of these studies unable to provide any substantial conclusions with respect to the common cold and other proposed health effects of vitamin C supplementation. To resolve the controversies, a modern approach of evaluating rigorously designed, mechanism-based studies is necessary.

#### 2.3.4. Supplementing C: From Formulation to Dose

Vitamin C formulations are as diverse as the recommendations and health claims made in support various marketed supplements. However, there is no data to suggest that any formulation containing ascorbate, alone or in combination with bioflavonoids, liposomes, vitamin C breakdown products, or minerals, has any measurable effect on vitamin C bioavailability in humans. While there are claims that vitamin C from natural *versus* synthetic sources, there is little evidence to support this in human subjects [128].

Pharmacokinetic studies suggest the consumption of at least 200 mg/day of vitamin C in healthy young men and women results in plasma concentrations greater than 50 μM [105,106]. At this dose, nearly complete oral bioavailability is obtained, leukocyte saturation is achieved, and urinary excretion is minimized, suggesting a saturation point has been reached for vitamin C transport. A dose of 200 mg/day can be achieved through a diet rich in fresh fruit and vegetables. Although the limitations of extrapolating this data have been discussed, it has been suggested that 200 mg/day of vitamin C should be set as an optimal intake level for the general population [1]. As mentioned above, it is possible that increased intake of vitamin C is necessary for plasma saturation in some individuals, such as the elderly [101] or individuals with various genetic polymorphisms [103], which would likely necessitate the use of supplements.

Can a case be made for “megadose” levels of vitamin C, or those in excess of 1 g/day? As much as different authors have put forth their own perspectives on the subject, there are few RCTs examining the effect of large daily oral doses of vitamin C. Thus, it is difficult to speculate on the beneficial effect of such doses. Pharmacokinetic data show that daily oral doses of vitamin C up to 2.5 g/day result in higher plasma ascorbate levels than a 200 mg/day dose, despite decreased bioavailability and increased urinary excretion of vitamin C [105,106]; however, the differences are small and biologically most likely of little consequence. It has been speculated that high plasma ascorbate levels can be maintained by frequent dosing with ascorbic acid [129], but it unclear what health effect would be achieved by this regimen. To date, no papers have shown a benefit of achieving greater-than-saturation plasma levels in the long term, and this mechanism would need to be delineated before a recommendation could be made.

One possible effect of megadose levels of vitamin C may be in the gastrointestinal tract, where it may prevent the formation of carcinogenic *N*-nitroso compounds [130] and highly reactive electrophilic compounds from the diet, such as acrolein [131] or lipid peroxidation products [132]. An intriguing idea for a biological role of vitamin C in the digestive tract is an exploration of beneficial changes to microbiota populations in the gut. However, in all of these cases, studies have yet to be performed, currently making it premature to draw any conclusions about the beneficial effects of oral supplementation with high-dose vitamin C. Large doses of intravenous ascorbate are associated with anti-cancer effects [133] and represent a promising area of research in the future that should be a subject of future review.

## 3. Methods

### 3.1. Ascorbate in Cell Culture Media

Phosphate Buffered Saline (PBS) solution was prepared from PBS tabs (Sigma Aldrich, St. Louis, MO, USA) dissolved in double deionized, filter sterilized water. Hyclone RPMI 1640 (Thermo Fisher Scientific, Waltam, MA, USA) was either used alone or after overnight chelation with Chelex 100 resin and the addition of 1 mM diethylenetriaminepentaacetic acid (DTPA). PBS or media was placed in 6-well culture dishes in a cell culture incubator (5%/95%: CO_2_/air; 37 °C) or at room temperature. Low trace metal ascorbic acid (Sigma Aldrich, St. Louis, MO, USA) was dissolved at a concentration of 10 mM in deionized water before addition to culture dishes. At specified times, aliquots were removed and acidified in 15% perchloric acid (PCA) containing 5 mM DTPA for vitamin C analysis as described below. Cell culture media containing 10% FBS from different commercial sources (Hyclone, Thermo Fisher, Sigma or ATCC) or aliquots of FBS alone were also acidified with an equal amount of PCA, as described above for the analysis of ascorbate content. However, no ascorbate was detected unless ascorbate was added to these solutions.

### 3.2. Human Subjects and Blood Collection

Five subjects were recruited for blood collection as part of a larger study on vitamin C metabolism approved by the Institutional Review Board for the Protection of Human Subjects at Oregon State University. Subjects were healthy non-smokers taking no prescription or non-prescription drugs that might influence vitamin C metabolism. For the study comparing anticoagulants, blood was collected after an overnight fast in vacutainers containing no additives or sodium heparin, lithium heparin, K2 EDTA, K3 EDTA, or sodium citrate (all from Becton Dickinson, Franklin Lakes, NJ, USA). Immediately after collection, aliquots of blood were collected from vacutainers and centrifuged at 16,000× *g* for 1 min for the separation of plasma from red blood cells. Aliquots of plasma were immediately acidified in 15% PCA containing 5 mM DTPA and placed at 4 °C to limit ascorbate oxidation until analysis, performed immediately, as described below.

For samples collected for the analysis of vitamin C oxidation in plasma, blood was collected in sodium heparin vacutainers from subjects after an overnight fast. Vacutainers were centrifuged in an Allegra X-15R (Beckman Coulter, Brea, CA, USA) at 4000× g at 4 °C for the separation of plasma. Vacutainers were then treated in one of three protocols: Oxidation control samples (“Control”) were kept on ice with limited light exposure until the vacutainer was opened in a chamber previously purged with nitrogen gas. Plasma was removed and acidified in PCA with DTPA. Vacutainers prepared with the standard protocol (“Standard”) were kept at room temperature and opened in ambient air before plasma samples were acidified with PCA. Vacutainers exposed to the environment (“Exposed”) were opened under ambient air at room temperature and plasma placed in a clear 2 mL tube. Tubes containing plasma were then incubated at room temperature for two hours before acidification.

### 3.3. Vitamin C and Urate Analysis

Plasma, media, and FBS samples were prepared for ascorbate analysis using HPLC as previously described [134]. Briefly, acid extracts were diluted in sodium acetate/methanol/water mobile phase (0.3%/7.5%/92% w/v) containing Q12 ion pairing reagent and pH-adjusted with 2.58 M KH_2_PO_4_ buffer (pH 9.8). Samples were separated on Watters 2695 using an LC-8 column (Supelco) under an applied potential of 600 mV using an electrochemical detector (BSA). Under these conditions, both urate and ascorbate are resolved and quantified by comparisons to authentic standards with a detection limit of approximately 1 nM. For human plasma samples, urate values are used to normalize ascorbate values to minimize any variations within subject samples and to control for volume differences in vacutainer analyses. To obtain total ascorbate, samples were reduced with the addition of tris-2-carboxyethyl phosphine (TCEP) as described elsewhere [113]. Dehydroascorbic acid content is estimated by the difference between TCEP-reduced and standard preparations.

### 3.4. Statistics

Statistical analysis was performed by GraphPad Prism software, version 6 (GraphPad Software, Inc., La Jolla, CA, USA). Statistical differences between ascorbate samples were determined by two tailed ANOVA using a Tukey post-hoc analysis, with a *p*-value below 0.05 considered significant. Non-linear regression analysis was used to determine the half-life of ascorbate in PBS or media.

## 4. Conclusions

Performing successful research on vitamin C, especially in the context of human health, requires specific focus and attention to detail. Although this review touched on many of the aspects of cell culture, animal, and human studies that are complicated by the use of ascorbic acid, it is not a complete guide to performing vitamin C research. Each study design is unique, so it must be critically analyzed with respect to ascorbate chemistry and biology to best understand whether and how it will make a contribution in the overall field. As described above, the effects of ascorbic acid within a biological system are potentially multifold, requiring extreme caution in implementation and interpretation.

As we change our view of vitamin C research, we must first address the most prevalent issues in frequently used model systems. The combination of high oxygen tension and the presence of reactive transition metals lead to a rapid oxidation of ascorbic acid in cell culture. The rapid loss of ascorbate in culture media promotes a pro-oxidant environment with the generation of superoxide radicals, hydrogen peroxide, and hydroxyl radicals, as described above. Moreover, depriving cells of ascorbic acid for countless generations produces a cell culture environment that is no longer relevant to human health. Overall, steps must be taken to control ascorbate oxidation if vitamin C research is to continue in cell culture. The use of stable ascorbate derivatives, metal chelation, and low oxygen culture conditions show promise, but much additional work is needed. Understanding the effects of prolonged ascorbate deprivation of cells is also necessary to determine if certain cell lines must be eliminated from use with ascorbic acid.

The continued use of ascorbate-synthesizing animal models also complicates all studies on ascorbic acid *in vivo*. Currently, mouse and rat models that lack ascorbic acid synthesis are available, but show considerable differences from guinea pigs and humans in terms of ascorbic acid bioavailability and metabolism. For example, the apparent lack of intestinal absorption of ascorbic acid in rodent models does not appear related to the synthesis capacity, and must be considered in supplementation studies. Furthermore, the effects of vitamin C deprivation appear to have effects in one model system that are not recapitulated in others, although we do not know if strain differences or dietary factors may have influenced these observations. The use of animal models is especially troubling in light of genetic alterations in the regulation and expression of vitamin C transport proteins in animals without functional ascorbic acid synthesis. Further studies are required to determine if other genetic adaptations have occurred—specifically in primate evolution—that may determine the difference in response of animals and humans to vitamin C supplementation.

Currently, studies involving vitamin C consumption in human subjects are not held to a rigorous standard. Randomized controlled trials, normally considered the benchmark for determining the impact of a compound on human health, have been poorly designed in regard to the chemistry and biology of ascorbic acid. While remaining aware of the effects that different study populations may have on the outcome of vitamin C supplementation, there is a need to determine vitamin C status in subjects before, during, and after a supplementation trial. Evidence shows that methods to record vitamin C intake by using food frequency questionnaires or food diaries are insufficient means of achieving this goal. Therefore study design needs to include assessments of vitamin C status using measurements of plasma, and possibly tissue, ascorbic acid levels. Only with these more precise determinations can we begin to speculate on mechanisms of action and eliminate speculations surrounding the health effects of vitamin C supplementation. Furthermore, eliminating the technical issues surrounding the measurement of vitamin C in clinical settings are additional steps needed to preserve data integrity.

While all study designs deserve higher scrutiny, a note of caution must also be placed in the interpretation of vitamin C research. It is all too common for vitamin C to be assigned a role (as an antioxidant or a pro-oxidant, for instance) without any supporting evidence to validate those claims. It cannot be overemphasized that the primary role of ascorbic acid in biological systems is that of a reductant, and the most established health effects of this reductive power are related to ascorbate’s role as an electron-donating enzyme cofactor used, e.g., for pro-collagen, carnitine, and catecholamine biosynthesis [3]. Ascorbic acid has many additional roles in the body beyond these enzyme functions, for example antioxidant protection, and likely more roles will be elucidated in the future. With a critical evaluation of all potential roles of ascorbic acid, the borders of vitamin C research can advance with balanced, evidence-based approaches. Already, emerging roles for vitamin C in various hydroxylase enzymes have recently placed a focus on HIF-1α-dependent gene expression [41,43] and changes in histone and DNA methylation [135,136], suggesting vitamin C may regulate global changes in gene expression. Further innovation has been demonstrated in studies on vitamin C bioavailability [69,108], metabolomics of vitamin C deficiency [137], genetic variation of the vitamin C transporters [103], and intravenous vitamin C infusions in cancer therapy [138,139].

As vitamin C research progresses into the 21st century, it has become clear that much more work still lies ahead of us. The experimental faults, artifacts, and myths currently afflicting vitamin C research limit the impact of many studies, making their contributions to general knowledge of the biological roles of ascorbic acid unremarkable at best, and confusing and detracting from the real issues at worst. This can also stretch beyond the realm of laboratory research, as the persistence of poorly controlled studies within this field often undermines efforts in the medical community to recommend vitamin C as a safe, effective means of promoting health. If nothing else, it weakens efforts to fund additional, well-designed RCTs necessary to establish definitive health claims that are desperately needed. In light of these issues, we must increase scrutiny of vitamin C studies in the past as well as the present, holding them to a higher standard based on the evidence discussed here if we are to make a lasting contribution to our understanding of vitamin C’s impact on human health.
